# Maternal Administration of the CNS-Selective Sobetirome Prodrug Sob-AM2 Exerts Thyromimetic Effects in Murine MCT8-Deficient Fetuses

**DOI:** 10.1089/thy.2022.0612

**Published:** 2023-05-04

**Authors:** Víctor Valcárcel-Hernández, Marina Guillén-Yunta, Thomas S. Scanlan, Soledad Bárez-López, Ana Guadaño-Ferraz

**Affiliations:** ^1^ Department of Endocrine and Nervous System Pathophysiology, Instituto de Investigaciones Biomédicas Alberto Sols, Consejo Superior de Investigaciones Científicas (CSIC)-Universidad Autónoma de Madrid (UAM), Madrid, Spain.; ^2^ Department of Physiology and Pharmacology and Program in Chemical Biology, Oregon Health and Science University, Portland, Oregon, USA.

**Keywords:** thyroid hormones, Mct8, T3 analog, MCT8 deficiency, fetal, prenatal treatment

## Abstract

**Background:**

Monocarboxylate transporter 8 (MCT8) deficiency is a rare X-linked disease where patients exhibit peripheral hyperthyroidism and cerebral hypothyroidism, which results in severe neurological impairments. These brain defects arise from a lack of thyroid hormones (TH) during critical stages of human brain development. Treatment options for MCT8-deficient patients are limited and none have been able to prevent or ameliorate effectively the neurological impairments. This study explored the effects of the TH agonist sobetirome and its CNS-selective amide prodrug, Sob-AM2, in the treatment of pregnant dams carrying fetuses lacking *Mct8* and deiodinase type 2 (*Mct8/Dio2* KO), as a murine model for MCT8 deficiency.

**Methods:**

Pregnant dams carrying *Mct8/Dio2* KO fetuses were treated with 1 mg of sobetirome/kg body weight/day, or 0.3 mg of Sob-AM2/kg body weight/day for 7 days, starting at embryonic day 12.5 (E12.5). As controls, pregnant dams carrying wild-type and pregnant dams carrying *Mct8/Dio2* KO fetuses were treated with daily subcutaneous injections of vehicle. Dams TH levels were measured by enzyme-linked immunosorbent assay (ELISA). Samples were extracted at E18.5 and the effect of treatments on the expression of triiodothyronine (T3)-dependent genes was measured in the placenta, fetal liver, and fetal cerebral cortex by real-time polymerase chain reaction.

**Results:**

Maternal sobetirome treatment led to spontaneous abortions. Sob-AM2 treatment, however, was able to cross the placental as well as the brain barriers and exert thyromimetic effects in *Mct8/Dio2* KO fetal tissues. Sob-AM2 treatment did not affect the expression of the T3-target genes analyzed in the placenta, but it mediated thyromimetic effects in the fetal liver by increasing the expression of *Dio1* and *Dio3* genes. Interestingly, Sob-AM2 treatment increased the expression of several T3-dependent genes in the brain such as *Hr*, *Shh*, *Dio3*, *Kcnj10*, *Klf9,* and *Faah* in *Mct8/Dio2* KO fetuses.

**Conclusions:**

Maternal administration of Sob-AM2 can cross the placental barrier and access the fetal tissues, including the brain, in the absence of MCT8, to exert thyromimetic actions by modulating the expression of T3-dependent genes. Therefore, Sob-AM2 has the potential to address the cerebral hypothyroidism characteristic of MCT8 deficiency from fetal stages and to prevent neurodevelopmental alterations in the MCT8-deficient fetal brain.

## Introduction

Inactivating mutations in the *SLC16A2* gene, which encodes for the monocarboxylate transporter 8 (MCT8), lead to an X-linked rare disease, the so-called MCT8 deficiency or Allan–Herndon–Dudley syndrome (AHDS).^1–3^ MCT8 is the only transmembrane transporter specific for thyroid hormones (TH), transporting thyroxine (T4) and the genomically active form 3,5,3′-triiodothyronine (T3).^4^ AHDS patients exhibit a spectrum of symptoms that has been widened recently, which include profound neurodevelopmental delay, cerebral hypothyroidism that leads to motor and intellectual disability, and peripheral thyrotoxicosis leading to a plethora of clinical sequelae.^5,6^

Currently, MCT8-deficient patients have limited treatment options and, because of the clinical complexity of the syndrome, with both cerebral hypothyroidism and peripheral hyperthyroidism, the designing of therapeutic strategies is challenging.^7^ Among those strategies that have been clinically tested, both TH replacement strategies, such as the combination of PTU and levothyroxine, and TH analog strategies, including diiodothyropropionic acid and triiodothyroacetic acid (TRIAC), have been able to ameliorate key features related to peripheral hyperthyroidism. However, none of them have been able to alleviate neurological outcomes up to date, even though there is still an ongoing clinical trial (NCT02396459) assessing if TRIAC could be able to ameliorate these symptoms in the youngest patients.^8–12^

The brain barriers, and in particular the blood–brain barrier, have been identified as the main restrictions for TH entry to neural cells in the absence of MCT8.^13–15^ For this reason, novel therapeutic strategies have been developed to overcome this limitation.^16–20^ Among these strategies, we previously obtained encouraging results using sobetirome and its pro-drug Sob-AM2. Sobetirome is a TH analog that binds both TH receptors with selectivity for the TH receptor beta over TH receptor alpha (TRA)^21,22^ and does not depend on MCT8 for its distribution in the brain. Sob-AM2 is a sobetirome amide prodrug that is converted into the active form sobetirome specifically in the brain by the action of the enzyme fatty acid amide hydrolase (Faah).^23^

For these previous preclinical studies, sobetirome and Sob-AM2 were tested in a validated murine model of the disease: the double knockout for MCT8 and deiodinase type 2 (DIO2, *Mct8/Dio2* KO).^24^ *Mct8KO* animals are only a partial model of the AHDS, as they replicate the endocrine but not the neurological alterations of patients.^25^ The lack of neurological alterations is due to a compensatory mechanism involving a larger presence of the T4 transporter organic anion-transporting polypeptide 1c1 (OATP1C1) at the mouse brain barriers in comparison with humans, and increased activity of DIO2, which converts T4 into T3.

Indeed, *Mct8/Dio2* KO and *Mct8/Oatp1c1* KO mice replicate both the endocrine and neurological alterations present in patients, thus have been extensively used as a model of AHDS.^24,26^ Systemic administration of sobetirome and Sob-AM2 to *Mct8/Dio2* KO mice was able to access the brain in the absence of MCT8 and modulate the expression of TH-target genes in juvenile mice.^18^ Because the neurological alterations in AHDS patients are already present from fetal stages,^27^ patients would benefit from prenatal treatment to prevent these defects. Indeed, some strategies have already evaluated maternal–fetal treatment for MCT8 deficiency^28,29^ both in humans and in murine models, with some positive therapeutic outcomes.

This study aimed to evaluate the ability of sobetirome and Sob-AM2 to cross the placental barrier and the fetal brain barriers to exert thyromimetic actions in the brain of MCT8-deficient fetuses as a potential therapeutic strategy to prevent neurodevelopmental alterations. To this aim, pregnant dams carrying *Mct8/Dio2* KO mice were treated systemically with sobetirome or Sob-AM2 and their fetuses compared with untreated *Mct8/Dio2* KO and wild-type (WT) fetuses. The results indicate that Sob-AM2 can cross the placental barrier and the fetal brain barriers, be converted in the brain into the active form sobetirome and exert thyromimetic effects in the brain of *Mct8/Dio2* KO mice in the absence of MCT8 at prenatal stages. Altogether, these data make Sob-AM2 a promising candidate to treat MCT8 deficiency from its most early stages.

## Materials and Methods

### Reagents

Drugs were prepared to be injected subcutaneously at 5 μL/g body weight concentration. Drug stocks from sobetirome (molecular weight [MV] = 328 g/mol; Sigma; SML1900) and Sob-AM2 (MW = 341 g/mol)^21,23,30^ were prepared by dissolving sobetirome and Sob-AM2 at 1 mg/mL in a previously prepared vehicle, composed of distilled water, Kolliphor^®^ (Sigma; C5135), and 1-Methyl-2-pyrrolidinone (Sigma; 328634) in 8:1:1 proportion. Saline was used to dilute the 1 mg/mL drug stocks to the final concentrations of 0.2 mg/mL sobetirome and 0.06 mg/mL Sob-AM2 (corresponding to 1.0 and 0.3 mg/kg dose, respectively). Since treatment with Sob-AM2 results in approximately twofold more sobetirome content in the brain than treatment with its precursor drug sobetirome,^18^ the chosen doses were 1.0 and 0.3 mg/kg of sobetirome and Sob-AM2, respectively, to achieve similar content of sobetirome in the brain with both treatments.

### Animal models and experimental design

**FIG. 1.**
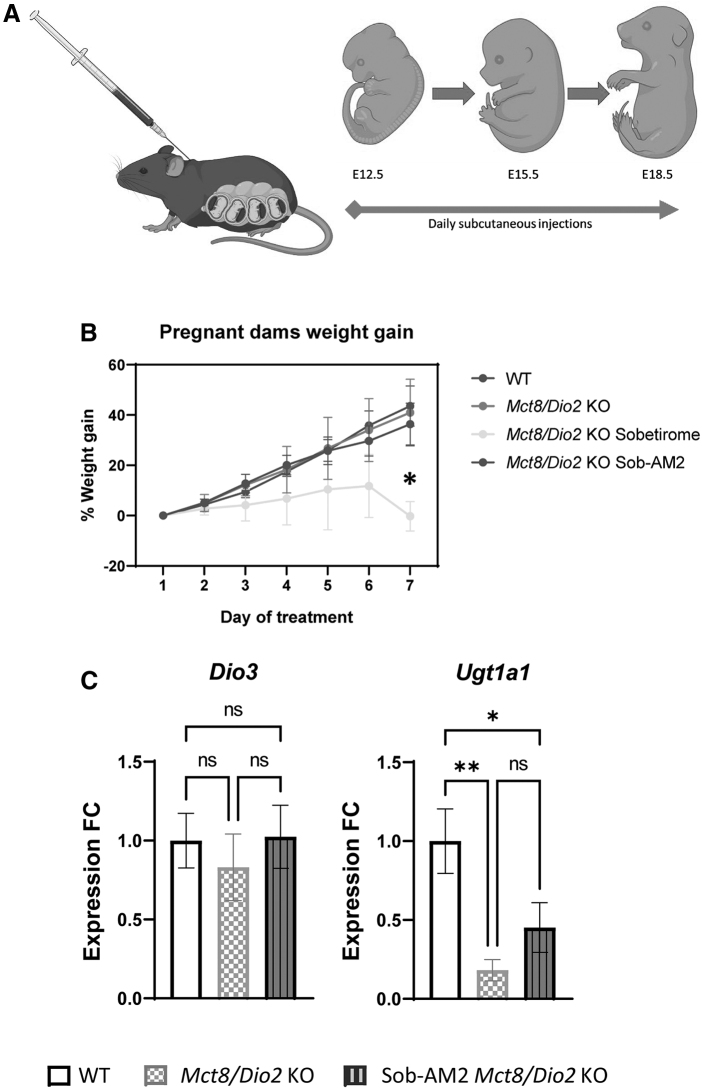
**(A)** Scheme representing the administration of TH analogs sobetirome and Sob-AM2 to pregnant dams. **(B)** Graph representing pregnant dams' weight gain during treatment (*n* = 5). **(C)** Graphs representing *Dio3* and *Ugt1a1* expression in the placenta in WT and *Mct8/Dio2* KO fetuses untreated and after Sob-AM2 treatment (*n* = 8 per group). Gene expression levels were obtained by qPCR, and the data are expressed relative to the average of housekeeping genes (18S, *Gapdh* and *Rplp0*) as fold changes of WT levels. Outliers identified with ROUT were excluded from the analysis. *p < 0.05, **p < 0.01 were determined by one-way ANOVA and Bonferroni's *post hoc* test. 18S, 18S ribosomal RNA; ANOVA, analysis of variance; *Dio3*, type 3 iodothyronine deiodinase; *Gapdh*, glyceraldehyde 3-phosphate dehydrogenase; qPCR, quantitative polymerase chain reaction; ROUT, Regression Outlier Removal tests; *Rplp0*, Ribosomal Protein Lateral Stalk Subunit P0; TH, thyroid hormones; *Ugt1a1,* UDP glucuronosyltransferase 1 family, polypeptide A1; WT, wild-type.

All mice were housed at the Instituto de Investigaciones Biomédicas “Alberto Sols” with *ad libitum* access to food and water, at 22°C and on a 12:12 diurnal cycle. *Mct8/Dio2* KO mice colony was previously established as described in Bárez-López et al.^24^ and mice were genotyped for *Mct8* and *Dio2* genes as described in Ceballos et al.^14^ and for fetal sex determination as described in Tunster.^31^ *Mct8/Dio2* KO breeding was done by mating M*ct8^+/y^ Dio2^−/−^* males and *Mct8^−/+^ Dio2^−/−^* females. The morning after mating was considered gestational day E0.5, then, pregnant WT females and *Mct8^−/+^ Dio2^−/−^* females (*n* = 5) were treated for 7 days (from E12.5 to E18.5, Fig. 1A) with daily subcutaneous injections at 5 μL/g body weight dosing.

Pregnant dams carrying *Mct8/Dio2* KO fetuses were treated with 1 mg of sobetirome/kg body weight/day, or 0.3 mg of Sob-AM2/kg body weight/day. As controls, pregnant dams carrying WT and pregnant dams carrying *Mct8/Dio2* KO fetuses were treated with daily subcutaneous injections of vehicle. Dams were euthanized using carbon dioxide asphyxiation at embryonic day E18.5, 4–6 hours after the last injection. Before fetal extraction, blood was extracted by retro-orbital collection and used for the determination of TH levels in maternal plasma. Then, fetuses were extracted into iced saline, and tissues (brain, liver, and placenta) were harvested for further analyses. Only male fetuses were analyzed as MCT8 deficiency is an X-linked pathology only expressed in males.

Animal experimental procedures were performed in strict accordance to the European Union Council guidelines (directive 2010/63/UE) and Spanish regulations (R.D. 53/2013). Approval for these procedures was granted by the ethics committee Comité de Ética y Experimentación Humana y Animal (CEEHA) at CSIC and by the Comunidad Autónoma de Madrid Review Board (Proex 014.1/21) for the use of animals for scientific purposes.

### Gene expression assays

RNA was isolated from individual hemi-cortex, liver, and placenta. Total RNA was extracted using TRIZOL reagent (Invitrogen; 15596026) following the manufacturer's instructions with an additional chloroform extraction. Real-time quantitative polymerase chain reaction (qPCR) was performed as described in previous publications.^32^

The expression of the following T3-dependent genes was measured using Applied Biosystems TaqMan probes. In the brain: *Dio3* (type 3 iodothyronine deiodinase), *Faah*, *Hr* (hairless), *Kcnj10* (Potassium Inwardly Rectifying Channel Subfamily J Member 10), *Klf9* (Kruppel-like factor 9), *Mgp* (Matrix gla protein), *Ncam1* (Neural Cell Adhesion Molecule 1), *Nrgn* (Neurogranin), and *Shh* (sonic hedgehog); in the liver: *Dio3*, *Dio1* (type 1 iodothyronine deiodinase), *Klf9*, *Ucp2* (uncoupling protein 2), and *Ugt1a1* (UDP glucuronosyltransferase 1 family, polypeptide A1); and in the placenta: *Dio3 and Ugt1a1*. The mean of the three housekeeping genes 18S ribosomal RNA (18S), Ribosomal Protein Lateral Stalk Subunit P0 (*Rplp0*), and glyceraldehyde 3-phosphate dehydrogenase (*Gapdh*) used as internal control to obtain relative mRNA expression. Data were expressed relative to the values obtained in vehicle-treated WT mice tissues (taken as 1.0).

### Maternal plasma TH levels determinations

Maternal plasma TH levels were determined using Biovision mouse T4 and T3 ELISA Kits (Biovision, Abcam, K7421, K7422) according to the manufacturer's instructions.

### Statistical analysis

Graphs represent mean ± standard error of mean. Normality of the data was assessed by the Shapiro–Wilk test. Differences between means were obtained by one-way analysis of variance (ANOVA) followed by Bonferroni's *post hoc* test to correct for multiple comparisons. Detection of outliers from a Gaussian distribution was performed by Regression Outlier Removal tests.^33^ Outliers were excluded from further analyses (see complete list at Supplemental Methods). All analyses were performed using the GraphPad software (GraphPad Software, Inc., La Jolla, CA).

## Results

### Sobetirome and Sob-AM2 effects in pregnant dams

Pregnant dams carrying WT or *Mct8/Dio2* KO mice were treated either with a vehicle, sobetirome, or Sob-AM2 from E12.5 to E18.5 (Fig. 1A). Sobetirome was observed to exert deleterious effects on sobetirome-treated dams' pregnancy. Weight gain of the pregnant dams was studied every day during the treatment, revealing that sobetirome-treated *Mct8^+/−^/Dio2^−/−^* dams weight gain became stagnant in the period between E15.5 and E18.5 (Fig. 1B), and during this period the treatment seemed to provoke spontaneous abortions.

This secondary effect, probably as a result of a peripheral toxicity effect associated with the dosage, resulted in the impossibility to reach an adequate sample number from sobetirome-treated dams. Sob-AM2-treated dams, however, did not exhibit this complication, as their weight gain was comparable with those of WT and vehicle-treated *Mct8^+/−^/Dio2^−/−^* dams (Fig. 1B) and they did not suffer deleterious effects and abortions during the treatment. In addition, there were no differences in the size of the litters (Supplementary Fig. S1A**),** or in the expected genotype and sex Mendelian distribution of offspring (Supplementary Fig. S1B, C) between vehicle- or Sob-AM2-treated *Mct8^+/−^/Dio2^−/−^* dams.

Plasma samples were then analyzed 4–6 hours after the last injection (E18.5). Maternal TH levels were assessed in nontreated and Sob-AM2-treated dams and the analysis revealed that *Mct8^+/−^/Dio2^−/−^* dams presented no differences in T3 and T4 plasma levels compared with WT dams (Supplementary Fig. S1D). Sob-AM2 treatment (once-daily, 7 days) in *Mct8^+/−^/Dio2^−/−^* dams decreased T4 plasma levels to ∼17% and 25% of the WT and *Mct8^+/−^/Dio2^−/−^* vehicle-treated dams, respectively, as previously observed in juvenile animals.^18^ T3 levels on Sob-AM2-treated dams, however, could not be assessed due to cross-reactivity between the treatment and the T3 ELISA. Thus, daily treatment with Sob-AM2 for 7 days resulted in depletion of circulating T4.

### Effects of Sob-AM2 in the placenta

The expression of two genes related to TH metabolism was analyzed in placental tissue. The expression of *Dio3,* responsible for metabolizing T4 into T3,^34^ was not altered in *Mct8/Dio2* KO placentas compared with WT animals (Fig. 1C). Treatment with Sob-AM2 did not change *Dio3* expression levels. The expression of the gene encoding the enzyme *Ugt1a1*, responsible for glucuronidation of both T3 and sobetirome,^35,36^ was decreased by 80% in the *Mct8/Dio2* KO samples compared with WT animals (Fig. 1C). There were no significant differences in *Ugt1a1* expression between basal and Sob-AM2-treated *Mct8/Dio2* KO fetuses, indicating minimal effects of Sob-AM2 in placenta.

### Effects of Sob-AM2 in the fetal periphery

**FIG. 2.**
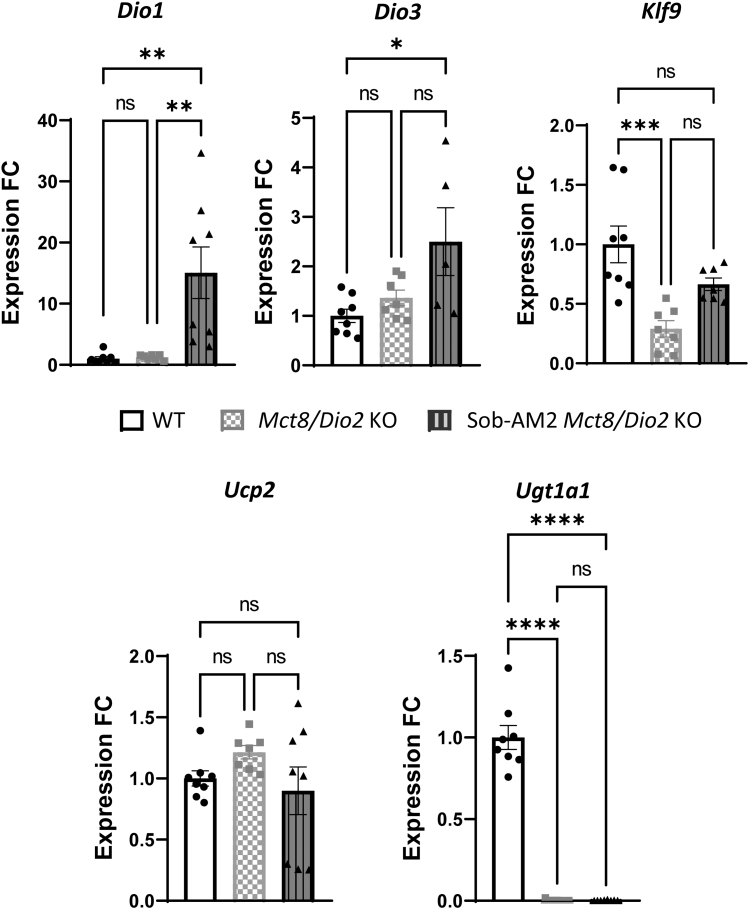
Graphs representing *Dio1, Dio3*, *Klf9, Ucp2*, and *Ugt1a1* expression in the liver in WT (*n* = 8) and *Mct8/Dio2* KO fetuses untreated (*n* = 7) and after Sob-AM2 treatment (*n* = 8). Gene expression levels were obtained by qPCR, and the data are expressed relative to the average of housekeeping genes (18S, *Gapdh* and *Rplp0*) as fold changes of WT levels. Outliers identified with ROUT were excluded from the analysis. *p < 0.05, **p < 0.01, ***p < 0.001, and ****p < 0.0001 were determined by one-way ANOVA and Bonferroni's *post hoc* test. *Dio1*, type 1 iodothyronine deiodinase; *Klf9*, Kruppel-like factor 9; *Ucp2*, uncoupling protein 2.

In the fetal liver, the expression of the T3-dependent genes encoding the TH metabolic enzymes *Dio1* and *Dio3* responsible for metabolizing T4 into T3^34^ presented no significant differences between *Mct8/Dio2* KO and WT fetuses (Fig. 2). Treatment with Sob-AM2 led to a 12-fold and 1.8-fold increase in the expression of *Dio1* and *Dio3*, respectively. The expression of the T3-dependent gene, *Klf9*, was 20% increased in *Mct8/Dio2* KO mice compared with WT fetuses and its expression was reverted to WT values in response to Sob-AM2 treatment. Expression of the T3-dependent gene *Ucp2* was not affected by any of the experimental conditions. The expression of *Ugt1a1* was also analyzed, showing a near 100-fold decrease in both treated and nontreated *Mct8/Dio2* KO fetuses as compared with WT.

### Effects of Sob-AM2 on the fetal CNS

Next, we evaluated the ability of Sob-AM2 pregnant dams' treatment to cross the placental barrier, reach the fetuses, and exert actions at the genomic level in the MCT8-deficient brain. T3-dependent genes were selected based on previous findings^18,37,38^ and included *Dio3*, enriched in neurons; *Hr*, enriched in oligodendrocyte progenitor cells (OPCs) and newly formed oligodendrocytes; *Kcnj10*, enriched in OPCs; *Klf9*, enriched in neurons, OPCs, and endothelial cells; *Mgp*, enriched in endothelial cells; *Ncam1*, enriched in neurons; *Nrgn*, enriched in neurons and OPCs; *Shh*, enriched in neurons and oligodendrocytes; as well as *Faah*, the enzyme responsible for converting Sob-AM2 into sobetirome, which is enriched in neurons and OPCs.^39^ Genes enriched in astrocytes and microglia were not included in the analysis, as these neural populations develop mainly at postnatal stages in the mouse.^40^

**FIG. 3.**
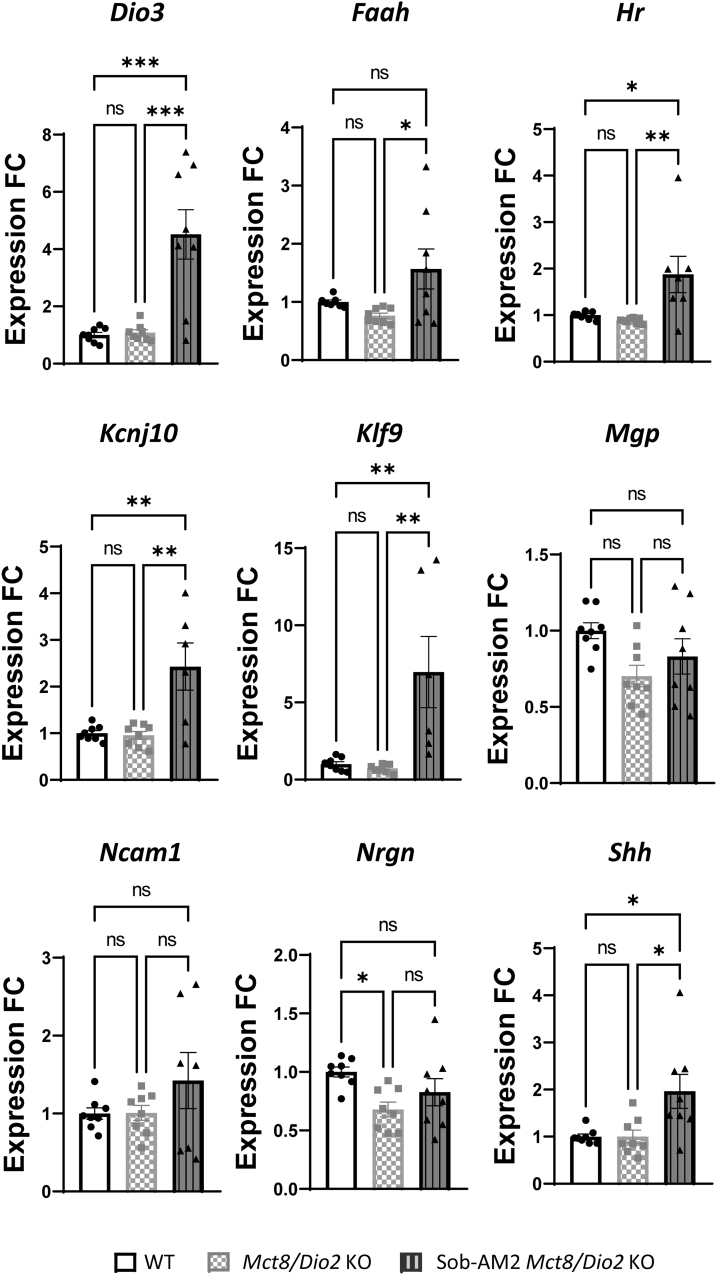
Graphs representing *Dio3*, *Faah, Hr*, *Kcnj10, Klf9*, *Mgp*, *Ncam1*, *Nrgn*, and *Shh* expression in the brain in WT and *Mct8/Dio2* KO fetuses untreated and after Sob-AM2 treatment (*n* = 8). Gene expression levels were obtained by qPCR, and the data are expressed relative to the average of housekeeping genes (18S, *Gapdh* and *Rplp0*) as fold changes of WT levels. Outliers identified with ROUT were excluded from the analysis. *p < 0.05, **p < 0.01, and ***p < 0.001 were determined by one-way ANOVA and Bonferroni's *post hoc* test. *Faah,* fatty acid amide hydrolase; *Hr*, hairless; *Kcnj10*, potassium inwardly rectifying channel subfamily J member 10; *Mgp,* matrix gla protein; *Ncam1*, neural cell adhesion molecule, *Nrgn,* neurogranin; *Shh*, sonic hedgehog.

Untreated *Mct8/Dio2* KO animals presented no significant differences in the expression of *Dio3*, *Faah*, *Hr, Kcnj10, Klf9, Mgp, Ncam1,* and *Shh*, and a 30% decrease in the expression of *Nrgn* compared with WT (Fig. 3). After treatment, the expression of all the studied genes in *Mct8/Dio2* KO was increased. *Dio3* expression exhibited a fourfold increase as compared with untreated *Mct8/Dio2* KO mice, *Hr* increased by 50%, *Kcnj10* by 60%, *Klf9* by 90%, and *Shh* by 50%.

Moreover, *Nrgn* expression levels were reverted in *Mct8/Dio2* KO mice after treatment, with no significant differences from WT values (Fig. 3). Sob-AM2 treatment also appeared to increase *Ncam1* and *Mgp* expression, although this effect was not statistically significant. In addition, *Faah* expression was analyzed, as this is the enzyme responsible for the conversion of Sob-AM2 to sobetirome in the brain. *Faah* expression was not altered between WT and untreated *Mct8/Dio2* KO mice but was significantly increased by 50% between nontreated and Sob-AM2-treated *Mct8/Dio2* KO fetuses, as a response to the access of Sob-AM2 into the brain.

## Discussion

This study explored the effects of the TH agonist sobetirome and its CNS-selective amide prodrug, Sob-AM2, in the treatment of pregnant dams carrying *Mct8/Dio2* KO mice fetuses. The main focus was to assess their ability to cross the placental barrier, and the fetal brain barriers, and reach the brain to exert a thyromimetic action in the absence of MCT8. In addition, it was important to analyze the treatment effects in peripheral tissues due to the complexity of the thyroidal status of this disease: peripheral hyperthyroidism versus cerebral hypothyroidism.

Our study revealed that Sob-AM2 (0.3 mg/kg) can exert thyromimetic actions on *Mct8/Dio2* KO fetuses when administered once daily for 7 days to pregnant dams. While this Sob-AM2 dosing schedule has already been shown to achieve therapeutic effects at postnatal stages,^18,41^ transplacental passage of this analog had not been demonstrated until now.

Sobetirome treatment resulted in failures during pregnancy and eventually spontaneous abortions, possibly due to hyperthyroid-like effects. Given that Sob-AM2 did not produce this deleterious effect and that Sob-AM2 can reach the brain almost twofold more than sobetirome, while being threefold less concentrated in the periphery,^18,42^ only treatment with Sob-AM2 was further evaluated.

Maternal Sob-AM2 treatment did not alter *Dio3* expression values in the placenta, suggesting a lack of Sob-AM2-derived sobetirome effects in this organ. However, mild upregulation of *Ugt1a1* could be indicative of sobetirome inactivation in the placenta. Sob-AM2 exerted thyromimetic effects in *Mct8/Dio2* KO mice fetuses. In the periphery, Sob-AM2 treatment increased the expression of both genes encoding the *Dio1* and *Dio3* enzymes in the liver, although it did not further increase the expression of the T3-target gene *Ucp2*, indicating a thyromimetic effect exerted by maternal Sob-AM2 treatment in fetal peripheral tissues.

Surprisingly, the expression of *Ugt1a1* levels in the liver of both nontreated and Sob-AM2-treated *Mct8/Dio2* KO fetuses was remarkably reduced in comparison with WT values, possibly by the lack of MCT8 and/or DIO2 interfering with other signaling pathways that regulate *Ugt1a1* expression, such as glucocorticoids.^43^ In the fetal central nervous system, increased expression of *Faah* following Sob-AM2 treatment strongly suggests that Sob-AM2 can access neurons and OPCs, neural cell types that express *Faah,*^39^ and enhance the activity of this enzyme to activate the conversion of Sob-AM2 into sobetirome. Moreover, the modulation of TH-target gene expression, which was able to normalize the expression of genes altered in nontreated *Mct8/Dio2* KO fetuses, demonstrates that maternal Sob-AM2 treatment can cross both the placental and the fetal brain barriers and induce thyromimetic effects in the fetal brain.

In addition, the data suggest that sobetirome derived from Sob-AM2 is able to exert actions in several neural cell types present at E18, as the modulated genes are expressed in neurons (*Dio3*, *Nrgn*, and *Shh*), OPCs (*Hr*, *Shh,* and *Kcnj10*) and endothelial cells (*Klf9*). However, it is important to mention that the expression of some TH-target genes have increased expression values in Sob-AM2-treated *Mct8/Dio2* KO fetuses over WT values, which could be indicative of a thyrotoxic effect at this dose. This is a proof-of-concept study that supports that Sob-AM2 can cross the placental and the fetal brain barriers and that can be converted into sobetirome in the fetal brain. In further studies, it would be of interest to assess the effect of maternal Sob-AM2 treatment on the thyroidal status of the fetus. Follow-up studies will shed more light into the ability of maternal treatment with Sob-AM2 to prevent the neurological alterations of *Mct8/Dio2* KO mice and will aid in identifying the optimum dose.

To sum up, our results indicate that a systemic Sob-AM2 administration to pregnant dams can cross the placental barrier and reach precisely the MCT8-deficient fetal brain to be subsequently converted into the active form sobetirome and exert thyromimetic actions in several neural cell types. As early treatment has been found to be crucial in AHDS clinical outcomes, the availability of a suitable TH analog that can target the fetal brain by maternal administration represents a great step forward in the possibility of treatments that prevent neurodevelopmental damage and alleviate the neurological symptoms of the disease.

## Supplementary Material

Supplemental data

Supplemental data
