# Supplementary material for: Maternal Administration of the CNS-Selective Sobetirome Prodrug Sob-AM2 Exerts Thyromimetic Effects in Murine MCT8-Deficient Fetuses
Source: Thyroid. 2023 May 4;33(5):632–40. doi: 10.1089/thy.2022.0612 (PMC10171952; doi:10.1089/thy.2022.0612)
Supplement: Supplemental data [file Supp_FigS1.docx]

**
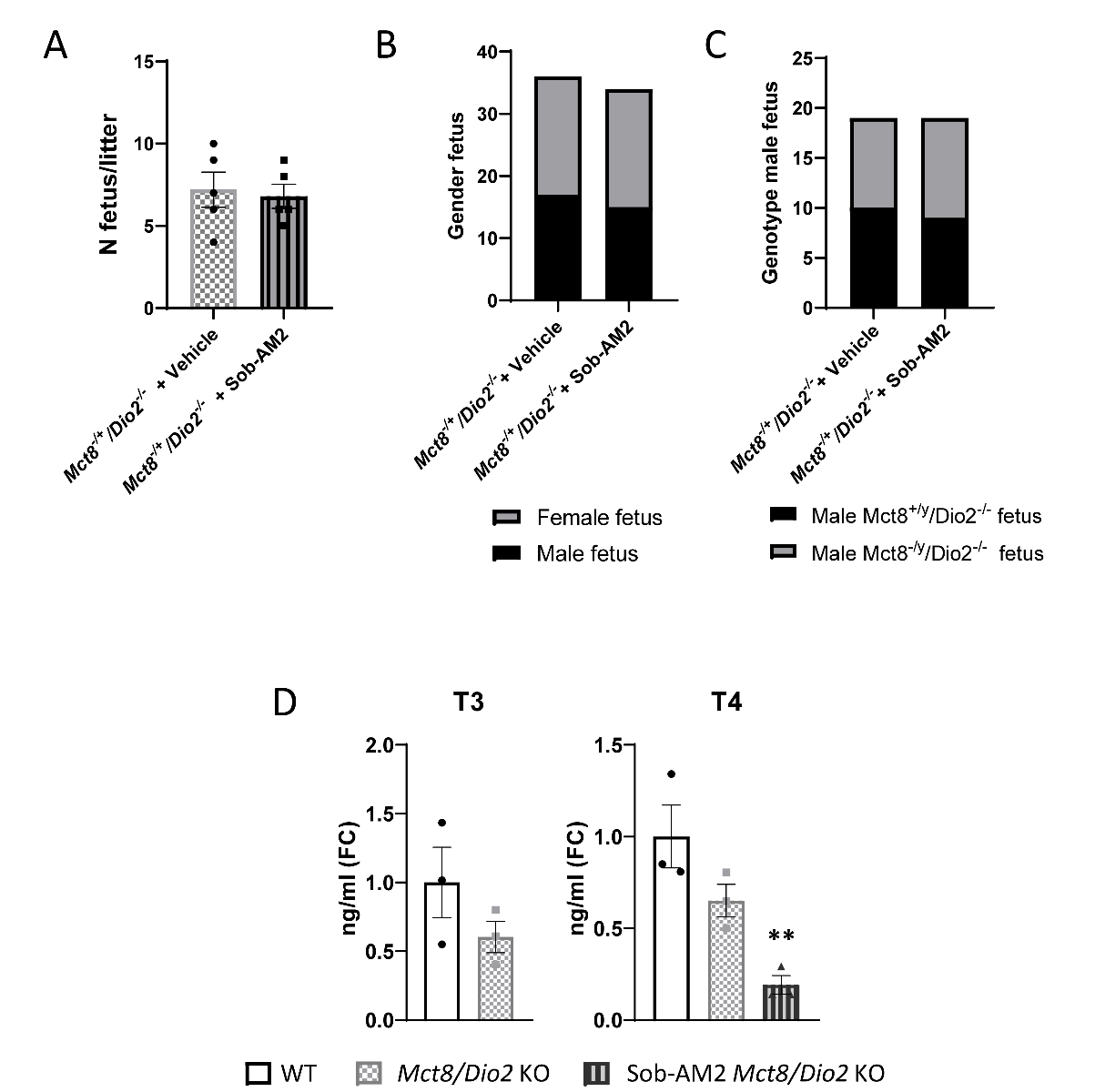
Maternal administration of the CNS-selective sobetirome prodrug Sob-AM2 exerts thyromimetic effects in murine MCT8-deficient fetuses: Supplementary material**

***SUPPLEMENTARY FIGURE 1***. (A) Graph representing the mean number of fetuses per litter (n = 5) ± SEM of *Mct8^-/+^/Dio2^-/-^* dams treated with vehicle or Sob-AM2, indicating no differences in the variance between both groups (F-test p = 0.8150). (B) Stacked bar chart from contingency table, indicating no differences in the expected sex mendelian distribution of the offspring of *Mct8^-/+^/Dio2^-/-^* dams treated with vehicle or Sob-AM2. (C) Stacked bar chart from contingency table, indicating no differences in the expected genotype mendelian distribution of the offspring of *Mct8^-/+^/Dio2^-/-^* dams treated with vehicle or Sob-AM2. (D) Graphs representing TH levels in plasma of WT and *Mct8/Dio2* KO carrying pregnant dams untreated and after Sob-AM2 treatment (n=3). The data are expressed relative to untreated WT carrying pregnant dams as fold changes of WT levels. **p < 0.01 differences *versus* WT group and were determined by Kruskal-Wallis test.
