# Supplementary material for: Maternal Administration of the CNS-Selective Sobetirome Prodrug Sob-AM2 Exerts Thyromimetic Effects in Murine MCT8-Deficient Fetuses
Source: Thyroid. 2023 May 4;33(5):632–40. doi: 10.1089/thy.2022.0612 (PMC10171952; doi:10.1089/thy.2022.0612)
Supplement: Supplemental data [file Supp_Data.docx]

SUPPLEMENTAL METHODS

1. **Study design:**
2. The groups compared were:

- In the thyroid hormone (TH) levels experiment: (1) WT dams treated with vehicle (control group), (2) *Mct8^+/-^ Dio2^-/-^* dams treated with vehicle and (3) *Mct8^+/-^ Dio2^-/-^* dams treated with Sob-AM2.
- In gene expression analyses: (1) WT fetuses treated with vehicle (control group), (2) *Mct8/Dio2 KO* fetuses treated with vehicle and (3) *Mct8/Dio2 KO* fetuses treated with Sob-AM2.

1. The experimental unit was single dams for TH measurements and single fetuses for gene expression analyses.
2. **Sample size:**
3. 5 dams per condition were used, for a total of 15 dams. This number of dams gestated the fetuses for the subsequent experiments, for a sample size of 8 fetuses per condition.
4. Sample size was calculated using the power calculation tool from the Experimental Design Assistant tool (EDA) of the National Centre for the Replacement Refinement and Reduction of Animals in Research (NC3Rs) (PMID: 28957312). We performed power calculations using previous findings where we had observed significant increases in the expression of TH-target genes following sobetirome treatment (for this purpose we have chosen *Abcd2*, PMID: 29845892). Using a significance threshold (α) of 0.01, a statistical power (1-β) of 0.95, introducing the largest variability (SD= 0.059) and an effect size of 0.1539 (increase in *Abcd2* expression in *Mct8/Dio2* KO animals treated with Sob-AM2 in comparison with *Mct8/Dio2* KO animals treated with vehicle), the recommended number of animals was n=8.
5. **Inclusion and exclusion criteria:**

a. Only males were used for the experiments as Allan-Herndon-Dudley syndrome is an X-linked disease. Outliers were identified by ROUT analysis and excluded from further tests.

b. All animals were included for every analysis apart from those identified as outliers by ROUT analysis. In TH levels, two samples per condition were not included due to technical reasons.

c. The exact value of n in each experimental group was:

Weight gain analysis: n=5 WT, n=5 *Mct8^+/-^ Dio2^-/-^*, n=5 *Mct8^+/-^ Dio2^-/-^* Sob-AM2.

T3 plasma: n=3 WT, n=3 *Mct8^+/-^ Dio2^-/-^*, n=3 *Mct8^+/-^ Dio2^-/-^* (Initial n=5, removal of 2 samples per condition).

T4 plasma: n=3 WT, n=3 *Mct8^+/-^ Dio2^-/-^*, n=3 *Mct8^+/-^ Dio2^-/-^* Sob-AM2 (Initial n=5, removal of 2 samples per condition).

Gene expression placenta n=8 WT, n=8 *Mct8/Dio2 KO*, n=8 *Mct8/Dio2 KO* Sob-AM2, except *Ugt1a1* WT n=6 (2 outliers), *Mct8/Dio2* KO n=7 (1 outlier).

Gene expression liver: n=8 WT, n=7 *Mct8/Dio2* KO, n=8 *Mct8/Dio2* KO Sob-AM2 except in *Ugt1a1* *Mct8/Dio2* KO n=5 (2 outliers) and *Dio3, Klf9* *Mct8/Dio2* KO Sob-AM2 n=5, n=7 (3 outliers and 1 outlier respectively).

Gene expression cerebral cortex: n=8 WT, n=8 *Mct8/Dio2 KO*, n=8 *Mct8/Dio2 KO* Sob-AM2. Except in *Hr*, *Ncam1 Mct8/Dio2 KO* Sob-AM2 *n=7* (1 outlier) and *Kcnj10, Klf9* *Mct8/Dio2 KO* Sob-AM2 n=6 (2 outliers).

1. **Randomization**

a. Dams were genotyped to distinguish *Mct8^+/-^ Dio2^-/-^* from WT. Once the genotype was identified, randomization to allocate treatment with vehicle or Sob-AM2 was done by the flipping of a coin.

b. Vehicle or Sob-AM2 treatments were alternated between each animal to minimize variability. Cage location was decided using <https://www.random.org/lists/>

1. **Blinding**

Researchers were not blinded during treatments. Researchers were blinded during RNA extraction and qPCR.
